# The effect of antifungal resistance development on the virulence of *Candida* species

**DOI:** 10.1093/femsyr/foac019

**Published:** 2022-03-23

**Authors:** Flora Bohner, Csaba Papp, Attila Gácser

**Affiliations:** HCEMM-USZ Fungal Pathogens Research Group, Department of Microbiology, Faculty of Science and Informatics, University of Szeged, Szeged, H-6726, Hungary; HCEMM-USZ Fungal Pathogens Research Group, Department of Microbiology, Faculty of Science and Informatics, University of Szeged, Szeged, H-6726, Hungary; HCEMM-USZ Fungal Pathogens Research Group, Department of Microbiology, Faculty of Science and Informatics, University of Szeged, Szeged, H-6726, Hungary; MTA-SZTE “Lendület” Mycobiome Research Group, University of Szeged, Szeged, H-6726, Hungary

**Keywords:** antifungal resistance, virulence, *Candida*, azoles, echinocandins, amphotericin B

## Abstract

In recent years, the relevance of diseases associated with fungal pathogens increased worldwide. Members of the *Candida* genus are responsible for the greatest number of fungal bloodstream infections every year. Epidemiological data consistently indicate a modest shift toward non-albicans species, albeit *Candidaalbicans* is still the most recognizable species within the genus. As a result, the number of clinically relevant pathogens has increased, and, despite their distinct pathogenicity features, the applicable antifungal agents remained the same. For bloodstream infections, only three classes of drugs are routinely used, namely polyenes, azoles and echinocandins. Antifungal resistance toward all three antifungal drug classes frequently occurs in clinical settings. Compared with the broad range of literature on virulence and antifungal resistance of *Candida* species separately, only a small portion of studies examined the effect of resistance on virulence. These studies found that resistance to polyenes and echinocandins concluded in significant decrease in the virulence in different *Candida* species. Meanwhile, in some cases, resistance to azole type antifungals resulted in increased virulence depending on the species and isolates. These findings underline the importance of studies aiming to dissect the connections of virulence and resistance in *Candida* species.

## Teaser

The authors discuss how the rising clinical relevance of fungal infections to the understanding of antifungal resistance development and its effects on the virulence attributes of pathogens could be highly beneficial, as it could lead to more efficient therapeutic practice.

## Introduction

Worldwide epidemiological data from recent years suggest that the relevance of fungal infections is increasing over time (Guinea 2014, Astvad *et al*. 2018, Goemaere *et al*. 2018, Lamoth *et al*. 2018). According to estimates, diseases associated with fungal pathogens are responsible for approximately as many fatal cases as tuberculosis and almost three times more than malaria annually (Bongomin *et al*. 2017, World Health Organization 2020a,b). Generally, *Candida* infections can range from mild superficial diseases—such as skin, hair and nail infections—to life threatening deep-seated manifestations, such as bloodstream infections. It is long established that species from *Candida*spp. are among the most prominent causes of fungal infections (Bongomin *et al*. 2017). Despite *Candida albicans* being the most frequently isolated species from patients with candidiasis, the relevance of non-albicans species is increasing globally (Guinea 2014). One of the reasons behind this distribution shift is hypothesized to be related to the characteristically diverse response to antifungals by different *Candida* species (Lortholary *et al*. 2011, Arendrup and Patterson 2017). For instance, one of the most frequently isolated non-albicans species, *C. glabrata*, is less susceptible to azoles than *C. albicans*. This trait confers a selective advantage to this pathogen in case of azole prophylaxis or treatment (Vale-Silva and Sanglard 2015). Furthermore, *C. parapsilosis* isolates have reduced susceptibility to echinocandins *in vitro*. Interestingly, the decreased echinocandin susceptibility of *C. parapsilosis* does not associate with unsuccessful treatment in clinical circumstances (Lortholary *et al*. 2011, Fernández-Ruiz *et al*. 2014).

Antifungal targets are limited due to the structural similarities of fungal and human cells. As a result, antifungal classes capable of efficiently treating systemic fungal infections are relatively low (Cowen and Steinbach 2008). As an effect of modern medical practice like prolonged parenteral nutrition or therapeutic immunosuppression, the size of the sensitive patient population shows a steady increase, whereas treatment options can be further limited by the appearance of antifungal resistance (Cowen and Steinbach 2008, Colombo *et al*. 2017, Sobel and Akins 2017). This observation further highlights the importance of understanding the mechanisms and effects of resistance, which can lead to more efficient therapeutic approaches. 

To underline the clinical relevance of understanding the connection between antifungal resistance and virulence, in 2020, 70 isolates of *C. albicans* isolated from patients with onychomycosis were studied in Iran. Results suggest that there is a positive correlation between increased azole (fluconazole, itraconazole) MICs and more pronounced biofilm formation as well as phospholipase production. The same pattern was identified between fluconazole resistance and hemolysin activity. These data indicate that the development of antifungal resistance could impact the expression levels of virulence factors (Mohammadi *et al*. 2020). A similar association was also found in the case of *C. glabrata* in a study that consisted of the characterization of isolates originated from Brazilian hospital units. The antifungal profile revealed that from the tested 91 isolates, 11 were MDR (multidrug resistant). A correlation was found between the secretion of virulence factors and antifungal resistance. In these strains, phytase production was connected with amphotericin B resistance and the secretion of esterase was linked with micafungin resistance. Additionally, the presence of hemolysin was associated with resistance to both fluconazole and micafungin (Figueiredo-Carvalho *et al*. 2017). To further strengthen the link between virulence and resistance, experimental studies found that the contact with innate immune system associated cells can promote the expression of characteristic genes involved in antifungal resistance, such as *CDR1* and *CDR2* in both *C. albicans* and *C. glabrata* (Fradin *et al*. 2007, Walker *et al*. 2009, Vale-Silva and Sanglard 2015). In some cases, it is more obvious how the selective pressure presented by antifungals has changed the epidemiological landscape of *Candida* species. In the last decades, vulvovaginal candidiasis (VVC) cases caused by *C. glabrata* have been rising steadily. To study the reasons behind the increasing incidence, Nakamura-Vasconcelos *et al*. used 16 *C. glabrata* strains, isolated from VVC infection. The selected isolates consisted of both fluconazole resistant (nine strains) and susceptible (seven strains) strains. Comparing the two groups, they found a positive correlation between fluconazole resistance and adherence efficiency as well as the biofilm forming ability of the strains. As a result, researchers hypothesized that the extensive use of fluconazole as a first line therapeutic agent and the subsequent increase of virulence capacity can be a factor in the emerging relevance of *C. glabrata* in VVC cases.(Nakamura-Vasconcelos *et al*. 2017).

Besides the presence of known virulence factors, microbial pathogenesis highly depends on general fitness attributes of the fungal strains; hence, the development of antifungal resistance often inevitably affects virulence (Ben-Ami *et al*. 2011, Lewis *et al*. 2012, Vale-Silva and Sanglard 2015). Depending on the *Candida* species and the utilized antifungals, these alterations can be both beneficial and disadvantageous to the fungal cells. Along with the increasing interest to understand mechanisms of antifungal resistance, several studies revealed direct or indirect connections between these processes and the pathogenic potential of the microorganisms.

In this review, our aim was to discuss how the development of antifungal resistance could impact virulence in clinically relevant *Candida* species, according to our current knowledge.

## Azoles

### Resistance mechanisms

**Figure 1.**
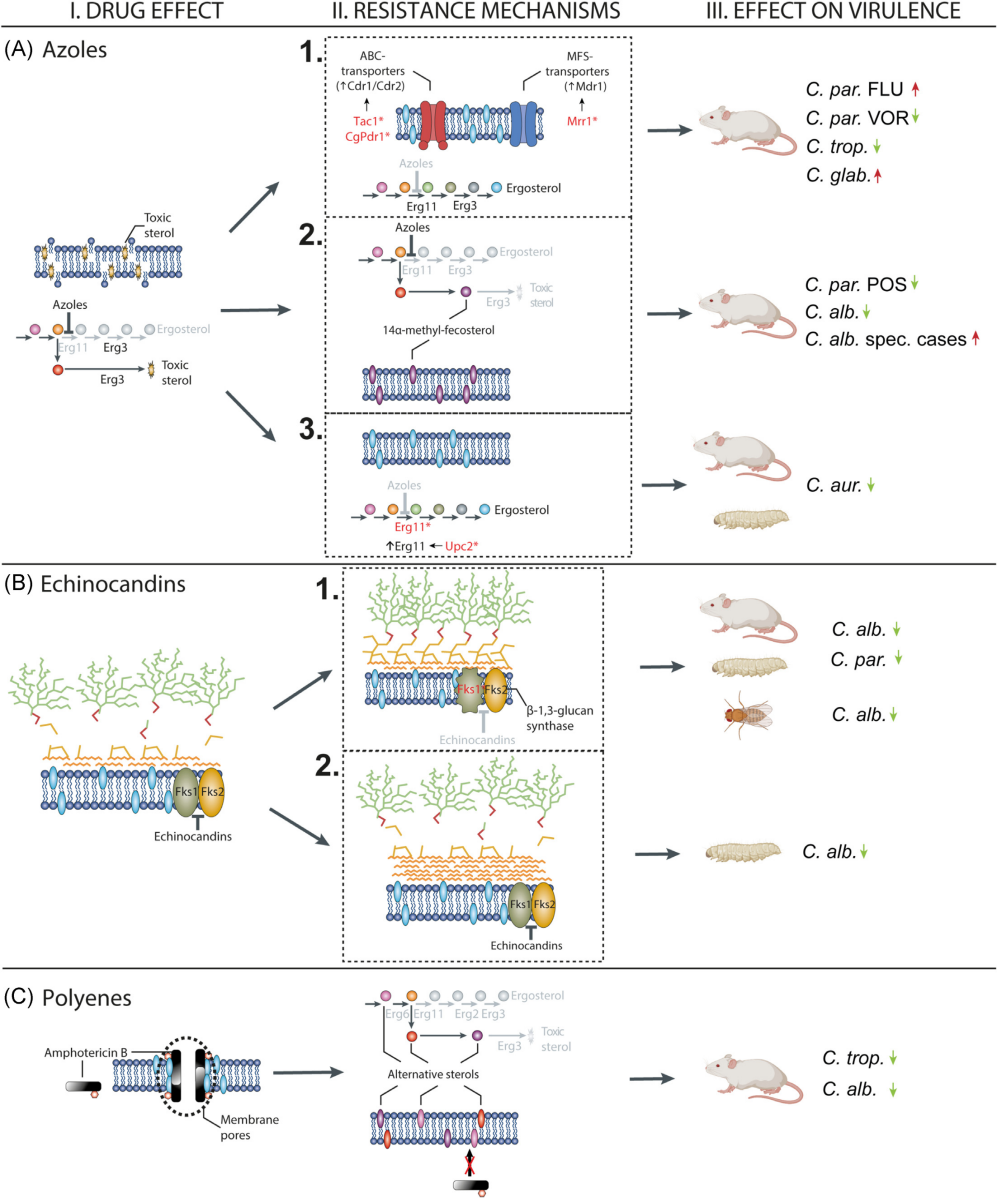
Effect of antifungal drugs (I), resistance mechanisms against antifungals (II) and the effect of resistance on virulence (III). Azoles inhibit the ergosterol biosynthesis in fungi, which leads to toxic sterol accumulation (I-A). Resistance against azoles can occur via efflux pump overexpression by transportation of drug molecules out of the cell (II-A/1), via *ERG3* loss-of-function (LOF) mutation leading to decreased toxic sterol production (II-A/2) or via mutation/overexpression of *ERG11* (II-A/3). The effect of overexpression of efflux pumps on the virulence is controversial as it caused increased virulence in *C. glabrata* and *C. parapsilosis* fluconazole-resistant strains but led to the reduction of virulence in *C. tropicalis* and voriconazole-resistant *C. parapsilosis* strains in mice model (III-A/1). In most of the studies, *ERG3* LOF mutation caused decreased virulence in posaconazole-resistant *C. parapsilosis* and *C. albicans* strains; however, in some special cases erg3 mutant *C. albicans* isolates showed increased virulence in mice (III-A/2). Overexpression and point mutation in *ERG11* caused decreased virulence in *C. auris* isolates (III-A/3). Echinocandins block β-glucan synthesis by inhibiting the Fks1p glucan-synthase subunit (I-B). Resistance to echinocandins can occur via point mutations in the *FKS*1 (II-B/1) or by the accumulation of chitin in the fungal cell wall instead of β-glucan (II-B/2). Both resistance mechanisms against echinocandins caused attenuated virulence in *C. parapsilosis* and *C. albicans* in mice, *Drosophilamelanogaster*, and *Galleria mellonella* models (III-B/1-2). Polyenes form pores on the plasma membrane on fungal cells as these antifungals couple with ergosterol, which causes cell death (I-C). Resistance to polyenes can occur by LOF mutations in different genes playing role in the ergosterol biosynthetic pathway that leads to accumulation of alternative sterols, which has no toxic effect on the fungal cell membrane (II-C). These alterations in the ergosterol biosynthesis pathway cause attenuated virulence in *C. tropicalis* and *C. albicans*, regularly (III-C). Figures were created using images of BioRender and our own graphical elements.

To this day, azoles are the most widely utilized antifungals due to their broad fungistatic activity, sufficient safety profile and relatively low price (Robbins *et al*. 2016, 2017). Azole type drugs are able to bind and inhibit the function of lanosterol 14-α-demethylase enzyme (encoded by *ERG11*), which results in the depletion of ergosterol. Additionally, the inhibition of *ERG11* activates an alternative ergosterol biosynthetic pathway, and subsequently the buildup of the toxic intermediate product 14-α-Me-3,6-diol (Fig. 1, I-A). These changes are rendering fungal cells unable to grow and divide while inducing membrane stress (Cowen and Steinbach 2008, Ostrosky-Zeichner *et al*. 2010, Shapiro *et al*. 2011). As expected, resistance to azoles is mainly associated with the modification of the ergosterol biosynthetic pathway. Point mutations in hot spot regions of *ERG11* have already been identified in resistant isolates of clinically relevant *Candida* species (*C. albicans*,*C. tropicalis*,*C. parapsilosis*,*C. glabrata*,*C. auris*). Such mutations (like Y132H, Y132F, K143R in *C. albicans*) lower the ability of azoles to bind to the target enzyme, decreasing the toxic activity of the antifungals (Xiang *et al*. 2013, Wang *et al*. 2015, dos Santos Silva *et al*. 2016). Another *ERG11* related mechanism is the overexpression of target enzymes. For example, in *C. albicans*, gain-of-function (GOF) mutations in the transcriptional regulator *UPC2* lead to constitutively higher expression of *ERG11* (Shapiro *et al*. 2011, Robbins *et al*. 2017) (Fig. 1, II-A/3). Functional loss of *ERG3* is also linked to azole resistance in *Candida* species, as following to the inhibition of *ERG11* by azoles, this enzyme is responsible for the synthesis of the toxic sterol product, which leads to changes of membrane permeability (Cowen and Steinbach 2008, Robbins *et al*. 2016, 2017, Healey *et al*. 2018) (Fig. 1, II-A/2). Besides to target alteration, the expulsion of the drug from the cell is a frequent way of *Candida* species to gain antifungal resistance. This process is associated with the overexpression of ATP-binding cassette (ABC) and major facilitator class (MFS) transporter proteins, such as Cdr1, Cdr2 and MDdr1. Gain-of-function mutations in the transcription activator *TAC1* are known to confer the constitutive upregulation of ABC transporters in several *Candida* species (Cowen and Steinbach 2008, Shapiro *et al*. 2011, Sanguinetti *et al*. 2015). In addition, similarly to *TAC1*, mutations in the regulator *MRR1* can be linked to antifungal resistance through the increased expression of *MDR1* (Fig. 1, II-A/1). Prominent presence of efflux pumps in the cellular membrane leads to a more effective elimination of the antifungals from the cytoplasm, which results in less azole susceptible cells (Morschhäuser *et al*. 2007, Robbins *et al*. 2017). Alteration of various stress response pathways following to antifungal treatment can also indirectly confer resistance. One of the key molecules, Hsp90 has a role in linking together several regulators of resistance. Such regulators include the protein phosphatase calcineurin, as well as the terminal mitogen-activated protein kinase (Mkc1) of the Pkc1 signaling cascade (Cowen and Steinbach 2008, Lafayette *et al*. 2010). Besides the Hsp90 chaperon, regulators of the target of rapamycin (TOR) pathway, were also shown to have a role in azole resistance. TOR pathway regulates cellular responses to environmental signals, such as changes in nutrient availability. These responses include several cellular processes from protein synthesis to lipid metabolism and cell wall integrity pathways (Hogan and Sundstrom 2009, Khandelwal *et al*. 2018). Genomic plasticity related sequence changes are also responsible for altered antifungal susceptibility. Development of aneuploidies as a response to environmental distress, like antifungal treatment, is a frequent adaptation mechanism among *Candida* species (Ahmad *et al*. 2014, Carreté *et al*. 2019). For example, the duplication of the left arm of chromosome 5 is rather common in azole-resistant clinical isolates of *C. albicans*. The formation of the isochromosome i(5L) leads to the increased dosage of *ERG11* and *TAC1* (Selmecki *et al*. 2006, Selmecki *et al*. 2008, 2009). Acquiring an extra copy of chromosome 5 following to antifungal pressure has also been associated with fluconazole resistance in *C. auris* (Bravo Ruiz *et al*. 2019). Furthermore, according to a study from 2009, examination of 40 *C. glabrata* clinical isolates originated from antifungal treated patients, two found to harbor an extra copy of chromosome E and chromosome D, respectively. Both duplicated regions contained known drug resistance associated genes, such as the ortholog of *Saccharomyces cerevisiae AUS1* and *PDR5* (Poláková *et al*. 2009).

### Effect of resistance on virulence and pathogenicity

LOF mutations in *ERG3* have been described as a classical mechanism conferring azole resistance in *Candida* species. As a result of these mutations, azole treatment cannot trigger the alternative pathway of ergosterol biosynthesis, thus the production of the toxic byproduct, 14-α-Me-3,6-diol (Cowen and Steinbach 2008, Ostrosky-Zeichner *et al*. 2010, Shapiro *et al*. 2011). Besides enabling resistance to antifungal drugs, *ERG3* mutations in *C. albicans* were linked to deficiencies in stress tolerance and hyphal growth, resulting in attenuated virulence in a C57BL/6 systemic mouse model. According to the results of *in vivo* experiments, despite the fact that *ERG3* mutant cells were able to persist within the tissues of murine organs, fungal strains were unable to cause lethal infections. In vaginal candidiasis models, the same *ERG3* mutant strains showed similar virulence attributes as the azole susceptible parental isolate, implying that certain mutations affect fungal cells in niche-specific ways (Luna-Tapia *et al*. 2018) (Table 1). Morio *et al*. studied two fluconazole, voriconazole and amphotericin B-resistant *C. albicans* strains with azole resistance conferring mutations in *ERG3*. Similarly to other studies, attenuated virulence of the resistant strains was noted in Swiss mice systemic infection model, hypothetically due to reduced filamentous growth capability (Morio *et al*. 2012). On the other hand, a work of Vale-Silva *et al*. suggests that in *C. albicans*, *ERG3* LOF mutations may be present, without the disturbance of normal hyphae formation. In their work, an azole-resistant clinical isolate (VSY2) that harbored a mutation in *ERG3* was capable of growing in filamentous form and consequently showed virulence attributes comparable to the parental strains in BALB/c disseminated candidiasis model (Vale-Silva *et al*. 2012). Taken together, these data and the scarce presence of *ERG3* mutant *C. albicans* strains in clinical settings imply that most of the fungal isolates harboring mutations in *ERG3* are unable to grow in filamentous form, which trait results in attenuated virulence. Nevertheless, the established existence of azole-resistant *ERG3* mutants with the ability to form hyphae to bypass decreased virulence leaves an alarming opportunity for the emergence of triazole-resistant *C. albicans* outbreaks (Martel *et al*. 2010, Vale-Silva *et al*. 2012) (Fig. 1, III-A/2).

**Table 1.** List of studies examining direct connections between antifungal resistance and virulence.

Mechanism of resistance/cellular process	Drug	Species	Effect	Reference
LOF mutation in *ERG3*	FLU	*C. albicans*	Reduced virulence in murine model of systemic infection; no change of virulence in murine model of vaginal infection	(Luna-Tapia *et al*. 2018)
*ERG3* mutations	FLU, VOR, AMB	*C. albicans*	Reduced virulence in murine model of systemic infection	(Morio *et al*. 2012)
LOF mutation in *ERG3*	FLU	*C. albicans*	No change of virulence in murine model of systemic infection	(Vale-Silva *et al*. 2012)
*MRR1* mutation	FLU	*C. parapsilosis*	Increased virulence in murine model of systemic infection	(Papp *et al*. 2020)
*ERG3* mutation (POS); *MRR1* (VOR)	POS, VOR	*C. parapsilosis*	Reduced virulence in murine model of systemic infection	(Papp *et al*. 2020)
*MDR1*,*CDR1* overexpression	FLU	*C. tropicalis*	Reduced virulence in murine model of systemic infection	(Barchiesi *et al*. 2000)
Overexpression of *CgCDR1* and *CgCDR2* as an effect of *CgPDR1* mutation	FLU	*C. glabrata*	Increased virulence in both immunocompetent and immunosuppressed murine model of systemic infection	(Ferrari *et al*. 2009)
*CDR1*,*CDR2* upregulation; mitochondrial disfunction (absence of *BPY41*)	FLU	*C. glabrata*	Reduced virulence in murine model of systemic and vaginal infection	(Ferrari *et al*. 2011a)
Hot spot mutations in *ERG11*; missense mutations in *CDR1*,*MDR1* and *TAC1*	FLU, AMB	*C. auris*	Attenuated virulence in *G. mellonella* model; reduced virulence in murine model of systemic infection	(Fan *et al*. 2021)
*FKS1* mutation	Echinocandins	*C. albicans*	Attenuated virulence in *Toll*-deficient *D. melanogaster* model; reduced virulence in murine model of systemic infection	(Ben-Ami *et al*. 2011)
*FKS1* mutation	CAS	*C. albicans*	Reduced virulence in murine model of systemic infection	(Wiederhold *et al*. 2011)
PG associated accumulation of chitin	CAS	*C. albicans*	Attenuated virulence in *G. mellonella* model	(Rueda *et al*. 2014)
*FKS1* mutation	AND, CAS, MICA	*C. parapsilosis*	Attenuated virulence in *G. mellonella* model; reduced virulence in murine model of systemic infection	(Papp *et al*. 2018)
Alterations of the ergosterol biosynthetic pathway	AMB	*C. albicans*	Avirulence in systemic murine infection model	(Vincent *et al*. 2013)
Alterations of the ergosterol biosynthetic pathway	AMB	*C. tropicalis*	Avirulence in systemic murine infection model	(Vincent *et al*. 2013)

In some cases, the development of antifungal resistance can have an incidental effect on several cellular processes. Secreted aspartic proteinases (SAPs) are recognized to be one of the most important hydrolytic enzymes playing a part in the virulence of *Candida* species. As a result, mutations in SAP genes cause attenuated virulence of *C. albicans* in mouse models (Naglik *et al*. 2003). Interestingly, several studies recognized that subinhibitory concentrations of certain antifungals (azoles, amphotericin B) can promote the production and secretion of virulence factors, such as SAPs (Fekete-Forgács *et al*. 2000, Mores *et al*. 2011). These findings present an indirect connection between the short-term response to drug treatment and proteolytic activity of the fungal strains, further underlining the importance of understanding cellular processes to improve therapeutic choices during fungal infections (Silva *et al*. 2014).

The effect of azole resistance on the virulence of *Candida* strains was also studied in *C. parapsilosis*. Long-term microevolution was used to generate resistant strains to fluconazole, posaconazole and voriconazole, respectively. Virulence changes of the evolved strains were studied in BALB/c systemic candidiasis model. Compared with the parental isolate, colonization of the kidney and brain by the microevolved strains was significantly altered. Voriconazole- and posaconazole-resistant strains colonized the kidneys less efficiently than both the wild type strains and the strains selected in the presence of fluconazole. Additionally, the fungal burden in the brain was noticeably higher in the case of the fluconazole evolved strain compared with the wild type, while the posaconazole evolved strain colonized this organ less efficiently. These data altogether suggest that virulence attenuation was the most prominent in the posaconazole evolved strain, while the fluconazole challenge was unable to cause the decrease of the pathogenic potential of *C. parapsilosis*. In line with the literature, *ERG3* mutation in the posaconazole evolved strain might be responsible for the severe virulence cost (Papp *et al*. 2020) (Fig. 1, III-A/1-2). To understand the development of fluconazole resistance in *C. tropicalis*, Barchiesi *et al*. also used an *in vitro* microevolution method to generate fluconazole-resistant strains. Further analysis of the generated resistant *C. tropicalis* strains revealed that the expression of both *MDR1* and *CDR1* positively correlated with the increase of MICs toward fluconazole. The generated resistant strain was found to be less virulent than the parental strain, as it was less successful to colonize the kidneys and failed to cause lethal infection in BALB/c mice following venous infection (Barchiesi *et al*. 2000) (Fig. 1, III-A/1).

In their study, Forastiero *et al*. used *C. tropicalis* clinical isolates with differing antifungal resistance profiles. One of the isolates ATCC 200956 showed cross-resistance to azoles and amphotericin B. Sequence analysis revealed major amino acid deletion in *ERG11*, rendering the protein nonfunctional, therefore the complete depletion of ergosterol was observed in this strain. In *in vivo* infection model, the resistant strain showed attenuated virulence in *G. mellonella* model compared with the susceptible isolate, highlighting the importance of the undisturbed sterol composition in the virulence of this species (Forastiero *et al*. 2013, Mesa-Arango *et al*. 2013).

In *C. glabrata* fluconazole resistance can be often traced back to the upregulation of ABC transporters such as Cdr1 and Cdr2. The role of the increased expression of these transporters is highlighted by the fluconazole hypersensitivity of mutants with disrupted *CDR1* and *CDR2* expression. Studying 122 *C. glabrata* isolates, Ferrari *et al*. found that azole resistance typically correlated with point mutations in the zinc finger containing transcription factor, *PDR1*. Further examination of this connection revealed that GOF mutations in three distinct ‘hot spot’ regions of *PDR1* can induce azole resistance by promoting the increase in the expression of *CDR1*,*CDR2* and occasionally *SNQ2*. To evaluate how mutations in *PDR1* affect the virulence of the isolates, both immunocompetent and immunosuppressed BALB/c mice models were used. According to their results, regardless of the utilized animal model, higher fungal loads were registered from the organs of mice infected intravenously with the azole-resistant isolate compared with susceptible strain. This data suggests that mutations conferring antifungal resistance can also be beneficial for the pathogen during host invasion (Fig. 1, III-A/1). It is important to note, that this *in vivo* fitness gain, associated with increased virulence, was absent in *in vitro* competition experiments, where *PDR1* mutants had no selective advantage compared with azole susceptible isolates (Ferrari *et al*. 2009). In a later study, Ferrari *et al*. found upregulated expression of both *CDR1* and *PUP1* (*PDR1* UPregulated gene) in *C. glabrata* strains harboring point mutations in *PDR1*. Constant overexpression of these genes in the more virulent *PDR1* GOF strains strongly indicates their connection to the enhanced pathogenic properties of these strains (Ferrari *et al*. 2011b).  According to the work of Moran *et al*., hyperactivation of *PDR1* could also partake in antifungal resistance by promoting the increased recruitment of the Mediator complex and other coactivators, to the promoters of transporter coding genes, such as *CDR1* and *CDR2*. Mediator, as an RNA polymerase II associated activator of transcription, has a role in the antifungal drug resistance in pathogenic fungi, while it also affects the virulence attributes of *Candida* species. The complex consists of several subunits, each responsible for distinct functions, therefore mutations in these subunits can impact genes responsible for the filamentous growth or biofilm formation of the pathogens, as well as the expression of efflux pumps. These findings indicate that Mediator could play a central role in connecting the acquisition of antifungal resistance to altered pathogenic potential in *Candida*spp. (Moran *et al*. 2019).  Ferrari *et al*. examined two sequential isolates of *C. glabrata*, both recovered from the same patient treated with fluconazole. According to antifungal tests, the earlier isolate showed low MIC to fluconazole, while the isolate recovered after fluconazole therapy was resistant. The azole-resistant strain showed the upregulation of ABC transporters, such as Cdr1 and Cdr2 and also exhibited mitochondrial dysfunction as a result of the absence of *BPY41* in mitochondrial DNA. Despite these mutations, the azole-resistant strain was able to colonize the host organism more efficiently in both systemic and vaginal BALB/c models, suggesting fitness gain despite severe respiratory deficiency.  Previous studies reported the presence of major virulence attenuation due to mitochondrial dysfunction. This data points out, that these mutations can positively and negatively affect virulence as well, depending on the fungal strains and experimental setup (Ferrari *et al*. 2011a). 

As mentioned above one of the well-known mechanism of antifungal resistance development is the emergence of aneuploidy under antifungal pressure. Rapid changes in the genomic organization in clinical *C. glabrata* isolates have been reported in many cases, suggesting that chromosomal rearrangements occur in high frequency in this non-albicans members of the genus (Poláková *et al*. 2009, Ahmad *et al*. 2014, Carreté *et al*. 2019). A study from 2009 analyzed *C. glabrata* isolates with additional small chromosomes and segmental aneuploidy of the left arms of chromosome E and F. Genome mapping revealed that several genes in this region have roles in the pathogenicity and antifungal susceptibility of *C. glabrata*. For example, duplicated segments of ABC transporter coding genes (*CDR1*,*PDH1*,*CAGL0F01419g* paralog of *S. cerevisiae AUS1*) were reported. Further analysis of the changes in chromosome E noted duplicated segments of virulence related genes (phospholipase B, extracellular glycosyl phosphatidylinositol-linked aspartyl proteases). They also identified a minichromosome derived from chromosome F, with a clear role in antifungal resistance. This minichromosome was stable in most cases under antifungal pressure and was lost in the absence of the drug. The presence of this drug responsive chromosomal dynamism indicates that in *C. glabrata* this mechanism may also have an essential role, linking pathogenic attributes and antifungal susceptibility, while similar broad genomic alterations could also have considerable importance in species frequently displaying high level of genome plasticity (Poláková *et al*. 2009).

Understanding the connection between antifungal resistance and virulence is especially crucial in the case of the recently emerging member of the *Candida*spp.,*Candida auris*. This species quickly became an alarming threat, mainly in clinical units, because of its remarkable ability to acquire resistance toward the most widely used antifungal drugs (Pristov and Ghannoum 2019, Chakrabarti and Singh 2020). Nonetheless, to this day the number of studies linking resistance to virulence is scarce in this emerging pathogen. A recent study from China compared two isolates, one of them susceptible to antifungals, while the other showed resistance to fluconazole and amphotericin B. Although the resistant isolate showed elevated SAPs production at higher temperatures *in vitro*, it was found to be less virulent than the susceptible strain in both *Galleria mellonella* and in systemic infection model (BALB/c) (Fan *et al*. 2021) (Fig. 1, III-A/3). It is also important to note, that the two studied isolates originated from different clades, making the direct comparison difficult due to the general variability of the pathogenic abilities of *C. auris* isolates from differing origins (Forgács *et al*. 2020).

When comparing studies, there are inconsistencies regarding the identified azole resistance mechanisms in different *Candida* species, which implies major variances in the process of resistance development even in a strain-dependent manner. Based on that we cannot rule out the clinical importance of nonconventional resistance mechanisms, especially when they are known to affect the virulence attributes of the pathogens. Stress response pathways have an essential role indirectly connecting the antifungal drug initiated cellular reactions to pathogenesis by regulating diverse processes. For example, Hsp90 manages cellular stress circuits by stabilizing several downstream proteins, such as calcineurin. Inhibition of this key chaperon reduces the azole and echinocandin resistance of *Candida* isolates (Robbins *et al*. 2017a). Similarly, calcineurin inhibition by cyclosporin A decreases fluconazole tolerance in resistant *C. albicans* isolates. The presence of calcineurin is also essential in response to other stress conferring agents, such as voriconazole, caffeine, calcofluor white and congo red. Furthermore, it also determines pathogenesis in mouse models, as calcineurin affects colony morphology and hyphal transition of fungal cells (Sanglard *et al*. 2003a). As a result of its pivotal role in antifungal resistance, targeted inhibition of calcineurin can be a potential option for improved antifungal therapy in the future. In 2019, Hans *et al*. through several experiments proved that magnesium deprivation affects the calcineurin pathway in *C. albicans* leading to increased susceptibility to fluconazole. Besides that, decreased virulence of *C. albicans* was also noted in magnesium restricted environment, hypothetically due to the disruption of hyphae formation, supporting the idea that magnesium chelation could improve the activity of membrane-targeting antifungals (Hans *et al*. 2019). It is also known that calcineurin and its potential target *Crz1* have an important role in the antifungal susceptibility and virulence of *C. glabrata* and *C. tropicalis* (Miyazaki *et al*. 2010, Chen *et al*. 2014).

Vacuolar proton-translocating ATPase (V-ATPase) also functions as a key factor in both antifungal resistance and virulence in *C. glabrata*. The deletion of *VPH2—*a gene that codes an assembly factor of the V-ATPases—confers decreased susceptibility of FLU (fluconazole), VOR (voriconazole) and AMB (amphotericin B), also responsible for reduced virulence in murine models of disseminated candidiasis. The reason behind the decreased virulence can be connected to the impaired vacuolar function of mutant strains that is responsible for increased susceptibility to environmental stress factors like the immune response of the host organism (Minematsu *et al*. 2019). 

## Echinocandins

### Resistance mechanisms

Echinocandins are the newest antifungal drugs, which were developed in the late ’90s. This group of antifungal drugs inhibits the catalytic subunit of β-glucan synthase enzyme (encoded by *FKS* complex), therefore perturbing the homeostasis of the cell wall (Fig. 1, I-B). Despite echinocandins having a relatively short application history in the clinical environment, infections caused by resistant *Candida* isolates regularly occur (Robbins *et al*. 2016). Generally, most of the clinically important *Candida* species are susceptible to echinocandins, however *C. parapsilosis* isolates bear the highest MIC values of this class of antifungals (Lortholary *et al*. 2011). Experimental data suggest that the development of echinocandin resistance in *Candida* species is starkly similar. Compared with azole resistance, target enzyme modifications are even more prominent in the case of acquired echinocandin resistance. Mutations in hot spot regions of the *FKS* complex coding regions are known to confer antifungal resistance by inhibiting the binding of the drug (Cowen and Steinbach 2008, Robbins *et al*. 2017a, Sobel and Akins 2017). Isolates harboring point mutations in *FKS1* and *FKS2* are frequently found in clinical settings, suggesting that these modifications have an *in vivo* role as they develop following to echinocandin therapy (Pham *et al*. 2014, Berrio *et al*. 2018, Chowdhary *et al*. 2018).       

### Effect of resistance on virulence and pathogenicity

The connection between virulence and echinocandin resistance seems more obvious compared with the data observed in the case of azoles. The main reason for that is that echinocandin resistance is usually associated with the mutations in certain hot spots of the *FKS1* gene (Fig. 1, II-B/1). As an effect of the deficient glucan synthesis and therefore the reduction of β-glucan in the cell wall, increased chitin content was noted as a compensatory mechanism. Several studies revealed how this compensatory effect relates to virulence changes. In 2011, Ben-Ami *et al*. conducted experiments with echinocandin-resistant clinical and laboratory *C. albicans* strains harboring homozygous mutations in their *FKS1* genes. Virulence properties of the strains were tested in *Toll-*deficient *D. melanogaster* model. In this model, the increase of the echinocandin MICs highly correlated with attenuated virulence compared with the susceptible wild-type strain. To confirm the virulence changes, the *FKS1* mutant strains were further tested in a systemic candidiasis model using BALB/c mice. Consistent with the results of the fly model, hypovirulence was also evident in mice infected intravenously with echinocandin-resistant strains. Phenotype analysis of the *FKS1* mutants revealed that both impaired growth rate and defective filamentation generally accompanied the reduced catalytic capacity of the glucan synthase complex. Additionally, increased chitin content in mutant cells can be linked to attenuated Dectin-1 mediated inflammatory response, and therefore decreased virulence (Ben-Ami *et al*. 2011, Lee *et al*. 2012). Similarly, Wiederhold *et al*. compared three *C. albicans* clinical isolates. One of them was susceptible to echinocandins, while the other two strains—harboring *FKS1* mutation—were resistant to caspofungin treatment. They found that the isolate with the lowest echinocandin MIC was the most virulent in a murine model of invasive candidiasis. While the two *FKS1* mutant strains showed differences in pathogenicity, they were both less capable of causing lethal infections than the susceptible isolate (Wiederhold *et al*. 2011).

In response to echinocandin therapy, several *Candida* species are capable of paradoxical growth (PG) *in vitro*. Relevance of this phenomenon in clinical practice is still unclear, but it cannot be ruled out as a virulence modifying factor. Paradoxical growth was found to be associated with changes in phenotype, such as the increase of cell size, clump formation and defects in cell separation. Similarly, PG was also associated with the accumulation of chitin, but it was not sufficient for the cells to escape the fungicidal activity of caspofungin. Fungal cells showing PG upon challenge with caspofungin, were used to infect *G. mellonella* larvae. Forty % of the larvae infected with the CAS treated *C. albicans* cells survived the experiment, whereas without drug treatment 100% mortality was noted, suggesting attenuated virulence of PG strains. Further examination revealed that in the case of PG, hyphae formation of fungal cells was significantly lower than in untreated control cells. Further investigation of immune responses pointed out that compared with untreated cells, caspofungin challenged PG cells induced a significantly stronger pro-inflammatory response (TNF-α; IL-17, IL-12) and lower anti-inflammatory response (IL-10) in primary murine peritoneal macrophages. Besides the defective hyphae formation, alterations in the cell wall due to the increased chitin content can potentially explain the change of virulence properties in PG cells (Rueda *et al*. 2014) (Fig. 1, III-B/2). Chitin itself is known to have diverse immunoregulatory functions depending on its fragment length and interactions with fungal proteins (Ben-Ami *et al*. 2011, Lee *et al*. 2012). In addition, echinocandin treatment can also cause the unmasking of β-1,3-glucan, allowing more effective immune recognition by the host organism (Lee *et al*. 2012, Lewis *et al*. 2012).

The work of Choudhary *et al* further straightens the role of chitin as a key component linking virulence with echinocandin resistance in *C. glabrata*. It has been previously shown that phosphoinositide signaling pathways have a role in resistance development to azoles by regulating various cellular processes such as protein trafficking, autophagy, cell cycle progression and hyphal morphogenesis (Choudhary *et al*. 2019). Additionally, phosphatidylinositol-4-phosphate is vital for the trafficking of the major multidrug transporter Cdr1, to the plasma membrane (Ghugtyal *et al*. 2015). Another component of the pathway phosphatidylinositol-3,5-bisphosphate (PI(3,5)P2) kinase (Fab1) is necessary for the reorganization of the actin cytoskeleton and azole stress survival (Bhakt *et al*. 2018). Further two components of the pathway, Vac7 and Vac14 are responsible for the regulation of the protein synthesis and turnover of PI(3,5)P2, also partake in azole resistance by controlling the quantity of the protein in *C. glabrata* (McCartney *et al*. 2014). Upon deletion of *VAC7* and *VAC14*,*C. glabrata* cells showed growth defects, reduced stress tolerance and sensitivity to caspofungin. Mutant strains were also shown to have attenuated virulence in BALB/c mice as both were less able to colonize the kidneys of the animals after intravenous infection. The change in the pathogenic potential of the mutant cells can be traced back to the impaired trafficking of the cell wall proteins, resulting in the increase of the chitin content similarly to previous findings (Choudhary *et al*. 2019). In a study from Papp *et al. in vitro* microevolution was used for the generation of echinocandin-resistant *C. parapsilosis* strains. An echinocandin susceptible isolate was incubated in the presence of increasing concentrations of anidulafungin, caspofungin and micafungin respectively. The virulence of the generated resistant strains was evaluated both in *G. mellonella* and in a murine model (BALB/c) of systemic candidiasis. In both models, all three of the evolved strains showed attenuated virulence compared with the echinocandin susceptible initial isolate (Fig. 1, III-B/1). Further analysis of the cell wall of the evolved strains revealed severe alterations in the surface exposure of β-1,3-glucan and especially chitin compared with the parental strain. The altered structure of the cell wall was presumably due to the effect of *FKS1* mutations in all three of the evolved strains. This study further affirms that the acquisition of echinocandin resistance correlates with virulence attenuation in *Candida* species (Papp *et al*. 2018).

## Amphotericin B

### Resistance mechanisms

Despite polyenes having been in clinical use in the last 70 years, resistance to these agents is relatively rare. Polyenes, like amphotericin B, bind to ergosterol in the fungal membrane, forming channels that cause the disruption of the cellular homeostasis (Fig. 1, I-C). Recently, another mechanism of action was also noted in the case of amphotericin B. Polyenes may extract ergosterol from the membrane, forming sponge-like structures and disturbing normal membrane function (Kamiński 2014, Carolus *et al*. 2020). Additionally, amphotericin B can also induce an oxidative stress response and therefore the death of fungal cells (Belenky *et al*. 2013, Mesa-Arango *et al*. 2014). Despite their remarkable fungicidal effect, their use in the clinical setting is limited, due to the structural similarities between ergosterol and cholesterol. During the treatment of fungal infections with amphotericin B, nephrotoxicity in the host is a common side effect (Robbins *et al*. 2017b). Amphotericin B resistance is generally associated with alternations in the ergosterol biosynthesis pathway due to LOF mutations in certain genes encoding key enzymes of sterol synthesis. For example, combined loss of *ERG3* and *ERG11* function in *C. albicans* leads to the depletion of ergosterol, hence the inability of polyenes to attach to their target (Sanglard *et al*. 2003b, Cowen and Steinbach 2008, Vincent *et al*. 2013, Carolus *et al*. 2020). Similarly, combined alteration of *ERG11* and *ERG5*, as well as *ERG6* and *ERG2* also prompt amphotericin B resistance in *C. albicans* (Fig. 1, I-C). Compared with *C. albicans*, *Candida* species with alternative membrane sterol profiles, like *C. haemulonii* species complex, also show differing susceptibility to polyenes (Carolus *et al*. 2020). Interestingly, amphotericin B resistance is more common in *C. auris*, as ∼30% of known isolates were found to have reduced polyene susceptibility. The study of resistant strains originating from both the clinical setting and experimental microevolution revealed point mutations in *FLO8* that, among other downstream functions, regulate the expression of *ERG11*. Although no direct correlation was found between amphotericin B MICs and altered *FLO8*, the appearance of these mutations suggests a key role for this gene in polyene resistance development in *C. auris* (Carolus *et al*. 2020, 2021).  

### Effect of resistance on virulence and pathogenicity

The observation that amphotericin B-resistant *Candida* strains are rarely found in patients treated for fungal infections, suggests that the development of resistance to this drug either comes with fitness loss or attenuated virulence (Lewis *et al*. 2012, Robbins *et al*. 2017a). Adaptation to this antifungal drug might probably require the collective accumulation of several resistance mechanisms (alteration of ergosterol biosynthetic pathway, acquired resistance to oxidative stress), which can lead to reduced fitness therefore the decrease of pathogenic potential (Vincent *et al*. 2013). To further understand this phenomenon, Vincent *et al*. studied amphotericin B-resistant *C. albicans* and *C. tropicalis* strains, generated artificially by microevolution process. Whole genome sequencing of the generated stains pointed out alterations in the ergosterol biosynthetic pathway (*ERG2* and *ERG3/11* mutations) in both strains. Additionally, resistant strains were proved to be avirulent in systemic mouse infection models (BALB/c), due to failure in resisting the host environment and to successfully colonize the organs of animals (Fig. 1, III-C). The lack of ability to withstand the host immune response can be traced back to the constitutive activation of Hsp90-dependent stress response pathways, compensating the hypersensitiveness of the resistant strains to several host modeling stress conditions (Vincent *et al*. 2013). In *C. albicans*, the presence of enolase presumably represents a more indirect connection between amphotericin B resistance and virulence. *ENO1* null mutants, unable to catalyze the conversion of 2-phospho-d-glycarate to phosphoenolpyruvate, were found to be more susceptible to stress conferring agents, such as AMB, FLU, VOR and calcofluor white. The fact that these deletion mutants showed reduced hyphal growth and reduced virulence in the systemic candidiasis model (BALB/c) suggests that the unhindered progress of glycolysis and gluconeogenesis could also be an important link between antifungal resistance and virulence in *Candida* species (Ko *et al*. 2013) (Fig. 1, III-C).

## Conclusion

Understanding the virulence attributes of *Candida* species is an essential task for researchers due to the rising number of *candidaemia* cases worldwide (Guinea 2014, Astvad *et al*. 2018, Goemaere *et al*. 2018, Lamoth *et al*. 2018). In parallel with bacteria, the frequency of resistance development to antibiotic agents is also pronounced among fungal pathogens. Resistant isolates of the most relevant members of the *Candida* genus have already been identified to all three routinely used antifungal classes, namely azoles, echinocandins and polyenes. The low number of available antifungals further complicates the selection of the most suitable treatment option (Cowen and Steinbach 2008, Colombo *et al*. 2017, Sobel and Akins 2017). Besides limiting therapeutic choices, it cannot be ignored that the acquisition of antifungal resistance also affects the pathogen, often impacting several vital cellular processes. Generally, as pathogens use these changes as compensatory mechanisms during antifungal treatment, in the absence of the drugs they confer negative effects on the fitness of the fungal cells (Lewis *et al*. 2012, Sanguinetti *et al*. 2015). For example, the relatively low abundance of amphotericin B-resistant *Candida* isolates in clinical settings indicates that in these cases the fitness tradeoff is so severe that resistant cells are unable to effectively infect the host organisms (Lewis *et al*. 2012). The target protein of echinocandin type antifungals, Fks1, is an integral part of the fungal cell wall biosynthetic pathway. Since the most definite mechanism of antifungal resistance to echinocandins is the alteration of the target enzyme via point mutations in the hot spot regions of the coding gene, as a consequence of the impaired synthesis, cell wall alterations are expected (Cowen and Steinbach 2008, Robbins *et al*. 2017, Sobel and Akins 2017). Along with the decreasing content of β-glucan, the ratio of other components such as chitins is known to rise in resistant strains (Vale-Silva and Sanglard 2015). Chitin is recognized to have diverse immunoregulatory functions. The increase of this polymer in the fungal cell wall was previously identified as an anti-inflammatory signal associated with attenuated Dectin-1 mediated inflammatory response. That connection can explain the attenuation of virulence upon echinocandin resistance. It is also important to note that despite echinocandin resistance in most cases being caused by a single point mutation, analysis of the gene expression of the resistant isolates strongly suggests complex genome-wide alterations (Ben-Ami *et al*. 2011). Based on that the appearance of resistant isolates with increased pathogenic capabilities cannot be ruled out in the future. *Candida* species can acquire azole resistance in diverse ways. Similarly, to other antifungal classes, the resistance to azole type drugs is often correlated with the loss of fitness in *Candida* species, although several studies indicate, that even the increase of pathogenic capability is possible as a response to antifungal treatment. For example, in *C. albicans* certain resistance conferring *ERG3* mutations have been identified, that did not result in impaired filamentous growth. Fungal cells harboring this mutation were able to infect the host organism as successfully as nonmutant strains (Vale-Silva *et al*. 2012). The analysis of *CDR1* and *CDR2* overexpressing *C. glabrata* cells reached similar results. In this case, the presence of the resistance conferring mutation in *PDR1* even increased the virulence of the strains, in both immunocompetent and immunosuppressed murine models (Ferrari *et al*. 2009). In certain situations, resistance conferring changes can have niche-specific effects. For example, *C. albicans* cells with LOF mutation in the *ERG3* can display attenuated virulence in systemic infection models but be as capable as the nonmutant cells to infect vaginal tissue. Niche-specific effects can drive attention to the possibility that antifungal treatment can fundamentally modify the epidemiological distribution of *Candida* (Luna-Tapia *et al*. 2018).

Taken together, with the rising clinical relevance of fungal infections the understanding of the development of antifungal resistance and its effects on the virulence attributes of the pathogens could be highly beneficial as it could lead to more efficient therapeutic practice.
